# Draft genome sequence of *Streptomyces lavendulae* FRI-5, a *Streptomyces* hormone producer

**DOI:** 10.1128/mra.01452-25

**Published:** 2026-03-30

**Authors:** Kohei Ikesaka, Eiji Okamura, Shigeru Kitani

**Affiliations:** 1 International Center for Biotechnology, The University of Osaka 12936 , Suita, Osaka, Japan; 2 Department of Chemistry and Biological Science, College of Science and Engineering, Aoyama Gakuin University 196857, Sagamihara, Kanagawa, Japan; University of Manitoba, Winnipeg, Manitoba, Canada

**Keywords:** *Streptomyces*, genome, *Streptomyces *hormone

## Abstract

The production of secondary metabolites, including antibiotics, in the genus *Streptomyces* is controlled by *Streptomyces* hormones. We report here the draft genome sequence of *Streptomyces lavendulae* FRI-5, which synthesizes IM-2 as a hormone. Bioinformatic analysis revealed the presence of a large number of genes for the biosynthesis of secondary metabolites.

## ANNOUNCEMENT

Members of the genus *Streptomyces* are well known for their ability to produce a structurally diverse range of secondary metabolites. Autoregulators are one of the types of signaling molecules involved in the initiation of the secondary metabolism; autoregulators have been regarded as *Streptomyces* hormones due to their very low effective concentrations and the involvement of specific receptor proteins in mediating their actions (1, 2). *Streptomyces lavendulae* FRI-5 (MAFF 116015) was isolated from the soil from Osaka, Japan, in 1970 and was donated to NARO Genebank, Japan, by a chemical company as a D-cycloserine-producing strain (3). This strain produces the *Streptomyces* hormone IM-2, which controls the production of several compounds including D-cycloserine (4–6). Furthermore, we identified a diol-containing polyketide and a polyene macrolide antibiotic from the same strain (7, 8). These findings suggest that *S. lavendulae* FRI-5 has high potential for the production of useful compounds.

Genomic DNA was extracted from the mycelium cultured in medium B (4) (12 h, 28°C, and 120 spm) using the salting out procedure (9) by treatment with lysozyme, proteinase K, 10% SDS, and 5 M NaCl solution. The DNA quantity and quality were evaluated by the PicoGreen method using Victor 3 fluorometry (PerkinElmer). Library preparation and sequencing were performed at the Macrogen Corp. (Kyoto, Japan). A genomic DNA library was generated with the SMRTbell Template Prep Kit after shearing of genomic DNA to 20 kb and was then analyzed using the single-molecule real-time (SMRT) sequencing technology on a PacBio RS II system (Pacific Biosciences) using P6-C4 chemistry. The number of polymerase reads from one SMRT cell was 73,500, totaling 847 Mb, with an average length of 11,526 bp and a subread *N*_50_ of 16,782 bp. Subreads were extracted after adapter trimming and filtering based on a minimum subread length of 500 bp.

A *de novo* assembly was performed using the Hierarchical Genome Assembly Process v.3.0 (SMRT analysis software v.2.3.0), which included pre-assembly error correction steps where single-pass reads were mapped to long seed reads (minimum 6,000 bp) to generate high-accuracy consensus sequences. The assembly was further refined through polishing with the Quiver algorithm, yielding three non-circular contigs with an *N*_50_ value of 7,398,656 bp. The circularity was evaluated by identifying terminal overlaps. The final genome of *S. lavendulae* FRI-5 is 8.13 Mbp in size from three contigs of 7,398,656, 459,407, and 272,013 bp, with a G+ C content of 73.1% and an 88-fold sequence coverage of the genome. The annotation of *S. lavendulae* FRI-5 based on DFAST (10) identified 7,314 protein coding sequences, 88 tRNA genes, and 21 rRNA genes. The AntiSMASH 8.0 pipeline (11) predicted the presence of 40 full or partial BGCs, including clusters coding for the biosynthesis of IM-2 (12), indigoidine (13), lavendiol (7), and lavencidin (14). These findings indicated that *S. lavendulae* FRI-5 has the ability to synthesize structurally diverse compounds. Thus, the draft genome sequence will deepen our knowledge of secondary metabolism governed by *Streptomyces* hormones and enable us to identify cryptic useful compounds.

## Data Availability

The draft genome of *Streptomyces lavendulae* FRI-5 is deposited in DDBJ under accession numbers BAAJAM010000001, BAAJAM010000002, and BAAJAM010000003. Project data are available under BioProject PRJDB37918 and BioSample accession number SAMD01699465. The raw sequence data are available under DDBJ Sequence Read Archive accession number DRR794825.
